# Chemical cues of female fertility states in a non-human primate

**DOI:** 10.1038/s41598-019-50063-w

**Published:** 2019-09-23

**Authors:** Marlen Kücklich, Brigitte M. Weiß, Claudia Birkemeyer, Almuth Einspanier, Anja Widdig

**Affiliations:** 1 Research Group of Behavioural Ecology, Institute of Biology, University of Leipzig, Talstraße 33, 04103 Leipzig, Germany; 2 Research Group of Primate Behavioural Ecology, Department of Human Behavior, Ecology and Culture, Max-Planck-Institute for Evolutionary Anthropology, Deutscher Platz 6, 04103 Leipzig, Germany; 3 Research Group of Mass Spectrometry, Institute of Analytical Chemistry, University of Leipzig, Linnéstraße 3, 04103 Leipzig, Germany; 4 Institute of Physiological Chemistry, Faculty of Veterinary Medicine, University of Leipzig, An den Tierkliniken 1, 04103 Leipzig, Germany; 5 German Centre for Integrative Biodiversity Research (iDiv), Deutscher Platz 5E, 04103 Leipzig, Germany

**Keywords:** Behavioural ecology, Animal behaviour, Chemical ecology

## Abstract

An increasing number of studies suggest that olfaction is important for communication throughout the order of primates. Callitrichids, in particular, have well-developed olfactory systems and use anogenital glands to produce scent marks. Behavioural studies have shown that male common marmosets (*Callithrix jacchus*) distinguish between odours from the peri-ovulatory and luteal phase of females. However, large gaps remain in understanding the chemical underpinnings of olfactory cues. To investigate whether chemical cues vary with female fertility and reproductive quality, our study combined behavioural bioassays with chemical analyses of the anogenital odours of female common marmosets using gas chromatography-mass spectrometry. We found that cycle states, age and parity have an impact on chemical profiles and further identified affected chemical substances. Our results confirm and expand on previous behavioural evidence for cues of fertility. Our results indicate that cycle-related substances likely act as chemical cues. Males could use such olfactory fertility cues to optimize their mating effort and thereby increase their paternity certainty. This certainty could enhance paternal care for their infants. The results of our study open a promising avenue to find the metabolic pathways from which chemical cues of fertility arise and to unravel their importance during primate evolution in future comparative studies.

## Introduction

Females of many species signal their fertile phase as well as reproductive quality to attract preferred mating partners^1^ and, by doing so, enhance their reproductive success^2^. For example, in several taxa ranging from birds to non-human primates, visual, auditory and/or olfactory cues can depict information on body condition, parasite resistance or female ovulation^3–7^. Even in humans, considered to not advertise their fertile phase^8^, ovulation in women seems detectable by men via cues such as the symmetry of paired soft tissues (i.e. ears and digits^9^), ornamentation (i.e. choice of dress^10^), voice^11^ and body scents^8,12^. It was moreover suggested that scents could most reliably reflect the fertile state because hormones control the menstrual cycle and at the same time directly influence odours^13^.

For non-human primates, female fertility cues have been examined primarily for visual traits such as skin colouration (*Macaca mulatta*^14^) or anogenital swellings (*Papio anubis*^15^). Furthermore, female copulation calls are considered to provide honest information about fertility (*Macaca sylvanus*^16^). Olfactory cues were generally less studied at least in some primate families, although the existence of prominent visual and auditory traits does not predict the absence of olfactory cues^17,18^. On one hand, primate species can vary in their use of sensory modalities and on the other hand, stimuli from different sensory channels can co-occur as ‘redundant’ or ‘non-redundant’ multimodal signals^19^. Indeed, there is increasing evidence from behavioural bioassays (e.g. *Saguinus oedipus*^20^) as well as some chemical analyses (e.g. *Lemur catta*^21^) that scents encode information on fertility in several primate species. Traits such as rank, age and parity may contribute to the reproductive quality of female primates (reviewed in ref.^22^), whereby age and parity frequently are confounded. While it is known that rank (*Callithrix jacchus*^23^) and age (*Aotus azarae*^24^) influence the scent of female primates, the effect of parity on female scents, at least when being independent of age, is not yet known. Furthermore, the chemical changes that provide the basis for olfactory cues of reproductive quality are still largely unknown because of methodological difficulties in recording and quantifying olfactory cues^25^.

Common marmosets (*C. jacchus*) are highly attractive to study reproductive parameters conveyed via scent. Both sexes show scent-marking behaviour with marks composed of secretions from so called ‘specialised’ apocrine and sebaceous glands as well as ‘non-specialised’ skin glands, urine, genital tract secretions and faeces^23^. In addition to the main olfactory system, they have a well-developed vomeronasal organ and show hormonal and behavioural reactions to scents^26^. Common marmosets live in extended family groups with a dominant breeding pair and additional male and female helpers required for successfully raising offspring^27^. Ziegler *et al*.^26^ presumed that the pair and family bonding in common marmosets could be maintained by olfactory communication (at close distance). Indeed, males increase their mating behaviour during the fertile period of females despite the absence of visual signs, which strongly suggests that males detect scents to infer ovulation^28^. Several studies confirmed that males react to peri-ovulatory odours with greater amounts of investigatory behaviour, sexual arousal and mounts^23,29^, but also with neuroendocrine changes^30^ and activations of brain areas accountable for sexual behaviour^31^. Hence, females seem to provide an olfactory fertility cue which enables males to optimise their mating efforts and thereby could increase paternity certainty by mate guarding breeding females during their peri-ovulatory phase^28,32^. High paternity certainty could then enhance paternal care, which improves infant survival^32^ and therefore increases the reproductive success of both males and females. However, even in this well studied species, large gaps remain in understanding the chemical underpinnings of olfactory fertility cues which thus are the focus of the present study.

Our study combines behavioural bioassays and chemical analyses of anogenital odours of females using gas chromatography-mass spectrometry (hereafter GC-MS). We confirmed and expanded on previous bioassays^23,30^ to confirm that males distinguish different cycle states using odours, and predicted males to be more interested in scents from females in a peri-ovulatory phase than from females in a follicular or luteal phase. The second focus of the study was to identify chemical compounds that potentially constitute female fertility cues as well as other indicators of reproductive quality, i.e. age and parity. The identification of fertility-related substances eventually provides the opportunity to find the metabolic pathways from which they arise and subsequently to ascertain whether they are ‘signals’, whose emission has evolved for communicative purposes, or simple ‘cues’ (see ref.^19^). We therefore collected scent samples from the genital area of twelve female common marmosets of different ages and parity over their menstrual cycles. Chemical profiles derived from GC-MS analysis were investigated for changes over the menstrual cycle in their composition and overall similarity. We expected an independent effect on the chemical profiles for cycle states (i.e. follicular vs. peri-ovulatory vs. luteal), age and parity. The occurrence of cycle-specific substances can be expected especially during the peri-ovulatory phase, but also before and after ovulation following changes of cycle-associated hormones as found in blackbucks (*Antelope cervicapra L*.^7^). Based on studies carried out in great apes^33^, we expected the majority of age-dependent substances to have decreasing intensities with increasing age. Due to a lack of comparable studies, an increase or decrease of substance intensities was difficult to predict for parity. During gestation mammals start to produce ‘mammary chemosignals’, which seem to decrease near weaning^34^. However, longer-term changes in secretions could potentially result in substances which are only found in primi- and multiparous females. Such changes of chemical compounds with cycle states, age and parity could allow males to assess the reproductive fertility and quality of females and, accordingly, to adapt their mating behaviour and pair bonding, respectively.

## Methods

### Animals

The study was carried out with common marmosets (*Callithrix jacchus*) from a colony of the Faculty of Veterinary Medicine at the University of Leipzig (Germany). All animals lived in pairs or small family groups, were trained for regular handling and were fed predominantly with vegetables, fruits, gum arabic and a protein source such as meat or eggs^35^. The animal husbandry protocols were approved by the regional board of Leipzig (Reference Number: 24-9168.11/17/68). All experimental procedures were in agreement with the German Animal Welfare Act for the care and use of laboratory animals and were approved by the Regional Council of Leipzig (TVV 16/15). All methods were performed in accordance with the relevant guidelines and regulations.

Female common marmosets were sampled during three defined menstrual cycle phases: (1) follicular phase (samples within three to nine days before ovulation), (2) peri-ovulatory phase (samples from the day of ovulation and the two days before) and (3) luteal phase (samples three to 14 days after ovulation). To determine the state of the ovarian cycle, ultrasound examination (mostly after sampling) as well as hormone analysis (progesterone) were used as described elsewhere^36^. Common marmosets have a mean cycle length of about 28 days (range 24–30 days) with a mean follicular phase of nine days and a luteal phase of 19 days^37^. The studied marmosets were prostaglandin F_2α_-controlled (0.8 µg/animal), which induces luteolysis and growth of new follicles, resulting in a cycle length of 23 days on average. There were no other medical treatments of sampled animals during the course of sampling.

### Bioassays

To collect samples for bioassays, precleaned swabs (100% viscose from ebelin, DM; baked at 130 °C for 30 minutes) were rubbed over the genital area of females for 20 seconds during different menstrual cycle phases. Swabs were stored in cleaned glass vials sealed with Parafilm™ at −20 °C for a maximum of 13 days. Defrosted swabs (thawed two hours before odour presentation) were placed in stainless steel wire-mesh tea eggs (precleaned with 70% ethanol) and presented to male common marmosets within their home cages in the absence of their female partners. A person blind to the sample attributes hung two tea eggs onto the grid inside the home cage at a distance of 4 cm to one another. Gloves were worn during all sampling and swab handling procedures to prevent contamination with human scents. The behaviour of the male was immediately video-recorded for one minute after placing the samples and in the absence of any person. This recording time was set based on results of preliminary test runs during which the animals showed interest in the tea eggs with scent samples predominantly within the first minute.

We presented swab samples from eight female common marmosets to eight males. All females and males were adult, reproductively intact and lived in pairs with reproductively intact individuals. Females were aged 1 to 15 years (mean age ± SD of 8.8 ± 6.0 years), five of them were nulliparous, one primiparous and two multiparous. Samples were taken within one menstrual cycle of the females during three menstrual cycle phases: (1) follicular phase (one per female, N = 8), (2) peri-ovulatory phase (two per female, N = 16) and (3) luteal phase (one per female, N = 8). Males were aged 4 to 10 years (mean age ± SD of 8.7 ± 1.9 years).

Each male received two different two-choice-tasks in randomized order: scents of females in a follicular vs. a peri-ovulatory phase (N = 8) and scents of females in a luteal vs. a peri-ovulatory phase (N = 8), with the positions of the two respective samples randomized. The two samples presented to a male simultaneously within a given presentation were taken from different females rather than from the same female to avoid confusion when sniffing scents of one individual from two different time points at once. Each male received only scents from unfamiliar (i.e. no prior physical contact) extra-pair females and never the same donor female twice. Furthermore, combinations of female scents were randomized within the prerequisites of the test setup. Altogether, this setup allowed us to control for individual differences in odour profiles and individual preferences.

We first verified the suitability of this set-up for bioassays in common marmosets in an initial two-choice test in which males were simultaneously presented with a swab sample from a female in the peri-ovulatory phase and a blank swab, with positions following a randomized order. This test showed that males distinguished between swabs with scents from females and blank swabs, i.e. they inspected samples from females longer than blank samples (Wilcoxon signed-rank test: N = 8, median _female_ = 12.2 sec, median _blank_ = 7.4 sec, U = 34, P = 0.02).

### Samples for chemical analysis

The chemical analysis was done with a different sampling material than the behavioural analysis (above), because adsorption properties influence the usability of each sampling material in a context-dependent manner. Whereas swabs are suitable for the presentation of body odours to individuals (e.g. in humans^38^), thermal desorption (TD) tubes evoked no interest of animals in pilot tests (Kücklich *et al*., unpublished data) due to their efficient adsorption properties. Because of these properties, however, TD tubes were shown to be more sensitive for the analysis of chemical profiles than swabs^39^. Thus, we used TD tubes (stainless steel TD tube, 1/4 in. × 3 1/2 in., Supelco, Bellefont, USA) filled with two adsorbent materials (0.095 g Tenax TA and 0.21 g XAD-2, Sigma-Aldrich, Steinheim, Germany) to sample airborne odours from the animals. We used a combination of two adsorbents to provide a broader coverage of substances with respect to their volatility and polarity^39^. The adsorbents were separated from each other and secured in the tube with glass wool (2 mm, Supelco, Bellefont, USA) and tubes were closed with Swagelok Brass caps (1/4 in., Supelco, Bellefont, USA). TD tubes were conditioned in a TD Clean Cube device (SIM Scientific Instruments Manufacturer GmbH, Oberhausen, Germany) at a temperature of 190 °C for 120 min and 200 °C for 30 min under nitrogen flow. For sampling, a pump (BiVOC2, Umweltanalytik Holbach GmbH, Germany) was used to suck 6 L of air (flow: ~ 1.5 L/min; validated in^40^) through the TD tubes.

We collected scent samples from twelve female common marmosets (from which three also participated in the bioassays) aged 1 to 11 years (mean age ± SD of 6.3 ± 2.9 years). Seven of the females were nulliparous, three primiparous and two multiparous. Two females were full sisters of the same age (7 years, degree of relatedness, *r* ~ 0.5), another two females were full sisters of different ages (4 and 5 years, *r* ~ 0.5), and a further two females were a mother-daughter dyad (1 and 5 years, *r* = 0.5). All other dyads were not closely related (*r* ≤ 0.25). Sampling was carried out over one cycle for three females, over two consecutive cycles for three other females and over three consecutive cycles for another six females. For each cycle, we collected repeated samples per female and cycle phase, resulting in a total of 18 ± 7 samples per female (range 8–27). Overall, samples were collected over a sampling time bucket of sixteen months.

The samples were taken from near to the genital area (distance of approximately 1 cm) randomly from the left or right side. MK collected a total of 212 animal samples of three menstrual cycle phases: (1) follicular phase (N = 56), (2) peri-ovulatory phase (N = 104) and (3) luteal phase (N = 52). Additionally, 31 blank samples were measured from tubes either handled like all other tubes but without pulling air through it (N = 9) or with air sampled from the sampling room in the absence of animals before or after the sampling procedure (N = 22). Additional to MK collecting all samples, one of four assistants was present to handle the respective animal. The identity of the assistant was controlled for in our statistical models.

### GC-MS analysis

We used a GCMS-TQ8040 composed of a Gas Chromatograph GC-2010 Plus and a Triple Quadrupole Mass Spectrometer (Shimadzu, Kyoto, Japan) for GC-MS analysis. The samples were introduced by a thermal desorption system TD-20 (Shimadzu, Kyoto, Japan) with injection in split mode (split ratio 5), desorption of the samples at 250 °C for 8 min and a helium flow of 60 mL/min to a Tenax TA filled cold trap (−20 °C). By heating up the cold trap to 250 °C for 1 min, the samples were injected into the gas chromatograph through the opened outlet split at 140.2 kPa. Electron-impact ionization was performed at 70 eV and 220 °C with a scan range of *m/z* 30–300. We used two consecutively connected columns (Rxi-1ms: 30 m length, 0.25 mm ID, 0.25 μm film, and SGE Analytical Science BPX50: 2 m length, 0.15 mm ID, 0.15 µm film, Restek GmbH, Bad Homburg vor der Höhe, Germany) for one-dimensional chromatography with helium 5.0 as carrier gas at a flow rate of 1.58 mL/min. The initial temperature was 35 °C which was increased, after 0.5 min, by 6 °C/min until a final temperature of 320 °C was reached and held for 25 min.

### Data analysis

#### Bioassay

Bioassay videos were analysed using Solomon Coder v. 17.03.22 without knowledge about the presented sample attributes. M.K. coded the duration of investigating presented samples (i.e. sniffing, scent marking at samples) during the one-minute presentation time. Sniffing was defined as putting the nose closer than one centimetre to the tea egg and scent marking as rubbing genitals or chest against the tea eggs (cf.^18^). Licking at samples did not occur. In order to exclude an observer bias, seven of the 16 videos were additionally coded by a second observer and a two-way single measure intraclass correlation (ICC) was calculated using the R package ‘ICC’ v. 0.84.1^41^ to determine the inter-observer reliability. Consistency between both observers was excellent (ICC = 0.94, 95% confident interval: 0.82–0.98, p < 0.001).

#### Chemical analysis

We used a semi-automated procedure to process the chemical profiles. First, we used AMDIS v. 2.65^42^ to perform an automated signal deconvolution and peak picking resulting in a peak list for each sample. Afterwards, we matched all peak lists and detected repeatedly occurring peaks with a self-written R script (by Lars Kulik) which automatically groups peaks with similar retention times together into retention time ranges. We only considered retention time ranges for further analysis if they occurred in at least 14 out of all 212 samples (N = 284). For each of these ranges the R script automatically extracted the most prominent masses and we manually verified the specificity and consistency of these mass spectral patterns by visual inspection of mass spectra. In this manner, we assigned retention time (adjusted per batch) and the according substance-specific *m/z* for 274 retention time ranges (i.e. 10 substances could not be verified). Hence, each range presumably corresponded to one substance described by retention time and specific *m/z*, but not (yet) named, i.e. chemically identified by library search.

For these 274 substances, peak areas for the peak-specific *m/z* were determined using the program GCMS solution v. 4.20 (Shimadzu, Kyoto, Japan). To exclude substances not originating from the animals (but, e.g., from the sampling material), known contaminations from previous studies using the same TD tubes and analytical procedures were excluded^40^, reducing the dataset to 248 substances. Finally, peak areas of blank samples were compared to animal samples and substances were excluded if the peak area in the blank equalled or exceeded those in the animal samples. Thus, a total of 160 substances was considered in the final analysis. Prior to statistical analyses, we calculated the relative peak area (peak area divided by sum of all included peak areas x 100) for each substance and sample to account for the variance of the total sample intensities.

### Statistical analysis

All statistical tests were calculated in R v. 3.4.3^43^.

#### Bioassay

We used the non-parametric exact Wilcoxon signed-rank test (WRT) to compare investigation durations (i.e. sniffing and scent-marking) of males (N = 8) between the two respective menstrual cycle phases of donor females using the R package ‘exactRankTests’ v. 0.8–29^44^.

#### Similarity between chemical profiles

We used ‘analyses of similarities’ (ANOSIM) based on Bray-Curtis indices to determine whether profiles from the same menstrual cycle state (‘follicular’, ‘peri-ovulatory’, ‘luteal’), age (in years: ‘1’, ‘4’, ‘5’, ‘7’, ‘8’, ‘9’, ‘10’, ‘11’) or parity (‘nulliparous’, ‘primi-/multiparous’) of females (N = 212 samples from 12 females) were more similar to each other than from different cycle states, ages or parity. The similarity for each combination of sample dyads was calculated based on the log(x + 1) transformed relative peak areas. To control for repeated samples per female, we used a customized ANOSIM computing p-values after permuting the samples within an individual.

#### Factors influencing the chemical composition

We conducted two linear mixed models (LMM) using the package ‘lme4’ v. 1.1.11^45^ to compare the chemical composition of 212 samples between menstrual cycle states, age and parity of females (N = 12). Given the population structure of the study subjects within the facility, we were able to consider age and parity of females as independent predictors in the analysis. The analysis of chemical datasets brings along several challenges, for instance, pronounced differences in intensities of biologically relevant substances, environmental effects on sample intensity and complexity, and varying degrees of contamination due to animal handling, all of which may affect the relative composition of the chemical profile^46^. Appropriate data transformation can help to handle such problems. Thus, we fitted the model with two different responses: [1] normalised relative peak areas per substance which were centred to a mean of 0 and scaled to a standard deviation of 1 and [2] log(x + 0.001) and arcsine transformed relative peak areas per substance. The normalisation adjusts all substances to the same size, giving them the same importance in the analyses and focusing the analysis to substance changes between samples rather than in relation to the rest of the chemical profile. The log-transformation is used to achieve normally distributed data and to reduce the relative impact of abundant substances while maintaining relative differences in the abundance of substances within a sample^46^.

For both models, the multi-variate data matrix of samples (N = 212) and substances (N = 160) was vectorised^47^, resulting in 212 × 160 = 33,920 relative peak areas as response variable. Menstrual cycle state (three factor levels: ‘follicular’, ‘peri-ovulatory’, ‘luteal’), age in years (continuous from 1 to 11 years, z-transformed), parity (two factor levels: ‘nulliparous’ or ‘primi-/multiparous’), genital side (two factor levels: ‘left’ or ‘right’), timing of ultrasound examination (two factor levels: ‘before’ or ‘after’ sampling), and the presence of the assistants involved in animal handling (separately for each assistant: ‘assistant1’, ‘assistant2’, ‘assistant3’, ‘assistant4’; two factor levels: ‘yes’ or ‘no’) were fitted as fixed effects. Matrix rows and columns (samples and substances, respectively) were implemented as random effects to avoid pseudoreplication and heteroscedastic variance^47^. Additional random effects were female identity (ID, twelve factor levels), housing room (three factor levels: ‘room1’, ‘room2’, ‘room3’) and sampling batch (27 factor levels, i.e. the combination of female ID and the respective cycle number). Our actual test predictors were the random interactions of menstrual cycle state and parity with substance as well as the random slope of age within the random effect substance, which also allowed us to identify the substances which were most affected by the predictors as those showing the steepest slopes^40^. In addition, we fitted all random interactions which were biologically meaningful and possible to achieve more reliable p-values (see Table 1).

**Table 1:** Model parameters included in both linear mixed models.

Model parameters	Variables
Fixed effects	cycle state, age, parity, genital side, ultrasound examination, assistant1, assistant2, assistant3, assistant4
Random effects	sample (matrix rows), substance (matrix columns), ID, room, sampling batch
Random slope within substance	age
Random interactions within substance	cycle state, parity, genital side, ultrasound examination, assistant1, assistant2, assistant3, assistant4
Random interactions within ID	cycle state, ultrasound examination, assistant1, assistant2, assistant3, assistant4
Random interactions within housing room	cycle state, ultrasound examination
Random interactions within sampling batch	cycle state, ultrasound examination

In total, we included 33 predictor variables (fixed and random effects) per model which were based on 12 female marmosets sampled repeatedly during a total of 27 menstrual cycles, resulting in 212 samples with 160 substances per sample (i.e. 33,920 data points). For both models, we found no violation of the assumptions when checking for normal distribution, homogeneity of the residuals or when plotting the residuals against the fitted values. All variance inflation factors of the test predictors were below 1.5 (determined with the R package ‘car’,^48^, thus showing no indication of collinearity. To determine the significance of the full model, we compared it to a reduced model which lacked the random interactions/slopes of menstrual cycle states, age and parity within substance, using a likelihood ratio test (LRT^49^). If the full models revealed significance, we tested the individual test predictors by LRTs comparing the full models with reduced models lacking only the respective individual test predictors. Finally, we extracted the slope estimates of all substances for predictors significantly affecting the chemical composition. Substances were considered to be most affected by a given predictor if the absolute slope estimate was at least two standard deviations above the average absolute slope estimate^40^.

For each test predictor (cycle state, age, parity) within each model ([1] normalised and [2] log-transformed, respectively), we tentatively identified all most affected substances. Substance identification was implemented in the program GCMS Postrun Analysis v. 4.20 (Shimadzu, Kyoto, Japan) by searching the mass spectra (see Supplementary text files) within the NIST Mass Spectral Library (NIST14; National Institute of Standards and Technologies, Gaithersburg, MD, USA) under consideration of the elution order of the substances. Peaks with overlapping signals of other peaks were identified using background subtraction. Additionally, five of the substances were confirmed with reference standards. We identified the substance classes of all substances and classified them as ‘potentially endogenous’ (likely originate from animals), when described as originating from animals in previous animal studies and no exogenous main source (e.g. plants) is known. ‘Potentially metabolized’ substances are those which are reported for animals as well as exogenous origins (e.g. plants), as they could have potentially been metabolized by the animal or skin bacteria. Thus, their occurrence and change could be informative as well, because metabolic processes and bacterial composition may vary with individual traits of the host^50–52^. Compounds for which we could not find an indication for the origin in the literature were designated as ‘unknown’.

## Results

### Bioassay

**Figure 1:**
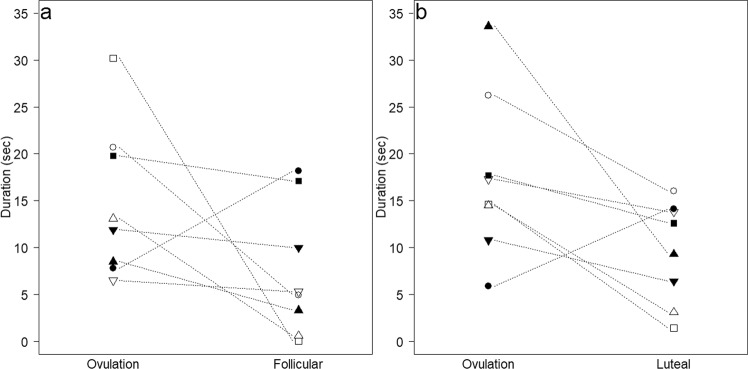
Duration of investigating simultaneously presented swab samples from female common marmosets from two different menstrual cycle states (**a**: peri-ovulatory vs. follicular and **b**: peri-ovulatory vs. luteal cycle state). Dotted lines connect data points from the same males that are also indicated by the same point characters.

Male common marmosets (N = 8) tended to investigate (i.e. sniff and scent-mark) swabs containing the odours of a peri-ovulatory phase longer than those of a follicular (WRT: median _ovu_ = 12.5 sec, median _foll_ = 5.1 sec, U = 30, P = 0.078, Fig. 1a) and a luteal phase (WRT: median _ovu_ = 16 sec, median _lut_ = 11 sec, U = 31, P = 0.055, Fig. 1b). All but one male clearly investigated the peri-ovulatory odours longer, while one male showed the opposite pattern in both trials (see Fig. 1: black circle).

### Similarity between chemical profiles

In the whole profile comparison of female common marmosets (N = 212 samples from 12 females), chemical profiles from the same menstrual cycle states tended to be slightly more similar than profiles from different cycle states (ANOSIM, R = 0.01, P = 0. 081) and profiles from the same ages were significantly more similar than profiles from different ages (ANOSIM, R = 0.32, P = 0.028). However, no difference in the similarity of chemical profiles was detected in association with parity (ANOSIM, R = 0.05, P = 0.435).

### Factors influencing the chemical composition

The chemical composition was significantly affected by menstrual cycle, age and parity of the females (N = 33,920 relative peak areas in 212 samples from 12 females), when substances were compared with each other between different samples (normalised model). When regarding substances relative to the whole sample (log-transformed model) only age and parity had a significant influence on the chemical composition (see Table 2). From the total of 160 substances comprising the whole data set, the differences in chemical composition were most pronounced in 31 ‘most affected substances’, i.e., their absolute slope estimate was more than two standard deviations above the average slope estimate (between six and eleven substances per predictor and model, see below).

**Table 2:** Results of likelihood ratio tests for all (full-null) and individual predictors including the number of most affected substances (N).

	Normalised model				Log-transformed model			
	*χ* ^*2*^	*df*	*p*	*N*	*χ* ^*2*^	*df*	*P*	*N*
Full-null model	195.05	11	<0.001		413.70	11	<0.001	
Cycle	4.23	1	0.040	6	0	1	>0.999	–
Age	130.79	1	<0.001	11	52.19	1	<0.001	8
Parity	24.54	1	<0.001	7	232.22	1	<0.001	10

### Substances changing between samples (normalised model)

**Figure 2:**
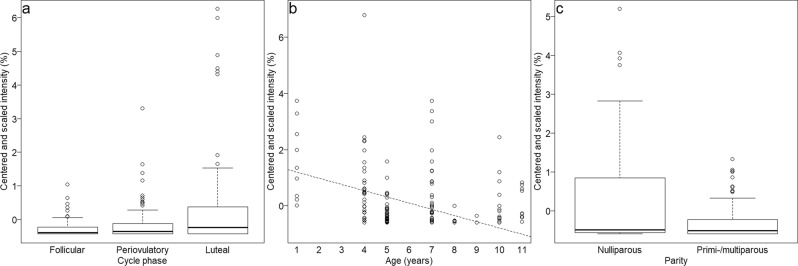
Influence of menstrual cycle state (**a**: 3-phenyl-1-butanol), age (**b**: 2,5-dimethyl-pyrazine) and parity (**c**: diisobutyl phthalic acid ester) on normalised intensities of the most affected substances. Boxplots show medians as well as first and third quartiles and the dashed line in scatterplot shows random slope estimates derived from a model with the fixed effects centred to a mean of zero.

Substances significantly varied relative to other samples with cycle state, age and parity of the females in the normalised model. Thus, their intensities changed relative to other samples, and animals would need to monitor the chemical composition over time. Six substances were most affected by cycle states (see Table 3). Four of those substances had the highest amounts during the luteal phase, including 3-phenyl-1-butanol that had the strongest cycle-dependent intensity change (see Fig. 2a). Two substances were most intense during the peri-ovulatory phase. With increasing age of the females, most of the eleven age-dependent substances (see Table 3) decreased, including 2,5-dimethyl-pyrazine, which showed the strongest change with age (see Fig. 2b). Only three substances increased with age. The seven most affected substances related to parity (see Table 3) had higher intensities in nulliparous than in primi-/multiparous females, for example, diisobutyl phthalic acid ester (see Fig. 2c), which had the strongest change in intensity.

**Table 3:** Most affected substances of both models with tentative identification (similarity: ^a^>900, ^b^>800, ^c^>700; ^d^confirmed with standard) and corresponding CAS-number, substance class (SC: *alco*hol, *alde*hyde, *alka*ne, *aro*matic hydrocarbon, carboxylic *acid*, *est*er, *het*erocyclic compound, *keto*ne, *pyr*azine, *ster*oid, *terp*ene), retention time (RT), indication of the origin (*pot*entially *end*ogenous, *pot*entially *met*abolised, *unkn*own as described in the methods section) and corresponding references.

Substance	CAS-Nr.	*SC*	*RT*	Normalised			Log trans		Origin
				Cycle	Age	Parity	Age	Parity	
Ethanol^d^	64-17-5	alco	2.4				2↓		pot met^69,70^
2-Pentanone^d^	107-87-9	keto	3.8		6↓				pot met^71,72^
3-Methyl-butanoic acid^a^	503-74-2	acid	6.8		9↓		5↓		pot met^59,71^
1,2-Dimethyl-benzene^a^	95-47-6	aro	7.8		10↓	6↓		3↓	pot met^69,70^
Heptanal^d^	111-71-7	alde	7.9		11↓				pot met^60,73^
3-Methyl-cyclopentyl acetate^a^	24070-70-0	est	7.9		3↓				unkn
2,5-Dimethyl-pyrazine^a^	123-32-0	pyr	8.1		1↓		1↓	4↓	pot met^70,74^
Benzaldehyde^d^	100-52-7	alde	9.2				4↓	6↓	pot met^73,74^
6-Methyl-5-hepten-2-one^a^	110-93-0	keto	9.9			5↓			pot met^59,75^
Phenylmethanol^a^	100-51-6	aro	11.2	5 O			3↓		pot met^71,75^
D-Limonene^a^	5989-27-5	terp	12.0			2↓			pot met^70,76^
1-Octanol^d^	111-87-5	alco	12.3			3↓		8↓	pot end^72,77^
Unknown aromatic hydrocarbon		aro	12.8		2↑				pot met^72^
Unknown aromatic hydrocarbon		aro	14.6	2 L					pot met^72^
Methenamine^a^	100-97-0	het	15.8					1↓	pot met^70,78^
Unknown		unk	16.3	4 O					unkn
Acetic acid linalool ester^a^	115-95-7	est	17.0		4↑				pot end^72,79^
1-Ethylidene-1H-indene^a^	2471-83-2	aro	17.2				7↑		pot met^80^*
Unknown alkane		alka	23.0		7↑				pot met^81^
2,6-Diisopropyl-naphthalene^a^	24157-81-1	aro	25.9					9↑	pot met^78^
Pyrrolidino[1,2-a]piperazine-3,6-dione^a^	19179-12-5	het	26.4	6 L					pot met^78^*
1,2-Diphenyl-1-isocyanoethane^b^	3128-88-9	aro	26.5				6↑		unkn
3-Methylbutyl benzoic acid ester^b^	94-46-2	est	27.8			4↓		7↓	pot met^82^
Benzoic acid, hexyl ester^c^	6789-88-4	est	28.3			7↓	8↑		pot met^78^
Diisobutyl phthalic acid ester^a^	84-69-5	aro	28.9			1↓		2↓	pot met^83,84^
2-Hydroxy-2-phenyl-ethyl benzoate^b^	10335-95-2	est	31.7	3 L					unkn
3-Phenyl-1-butanol^a^	2722-36-3	alco	31.9	1 L					pot met^72^
5-Isopropyl-2-methyl-phenyl benzoate^b^	100752-52-1	est	33.2					10↑	unkn
Unknown steroid		ster	43.1		8↓				pot end^72^
(3β)-Cholesta-4,6-dien-3-ol^a^	14214-69-8	ster	43.2		5↓				pot end^72^
Cholesta-3,5-dien-7-one^b^	567-72-6	ster	46.6					5↓	pot end^72^

For normalised and log-transformed models separately, substances are ranked by the strength of the effects in numbers within predictors (1 = most affected substance). Arrows and letters indicate the direction of the effect (↑ = increasing intensities with increasing age/parity, ↓ = decreasing intensities with increasing age/parity, L = intensities highest in luteal phase, O = intensities highest in peri-ovulatory phase).

Reference information: human skin^59^; human feces^69^; human feces, urine, breath, skin, milk, blood or saliva^70^; urine of *Peromyscus maniculatus*, ventral gland and sacculi of *Phodophus sungorus* or gland secretion of *Castor fiber*^71^; review of mammalian studies and report of rules to figure out substance origin^72^; sternal gland secretion of *Mandrillus sphinx*^73^; circumgenital scent secretion of *Callithrix jacchus*^74^; human skin emanation^75^; human faeces^76^; preorbital secretion of *Raphicerus campestris*^77^; ‘human metabolome database’: cytoplasm, extracellular space or cell membranes^78^; human axillary sweat^79^; human breath^80^; description of microbial biosynthesis of alkanes^81^; sweet cherry, papaya, quince, cherimoya vinegar, beer, cocoa^82^; urine in *Tupaia belangeri*^83^; human skin emanation^84^, *exact substance could not be found, but very similar structure.

### Substances with relative differences within samples (log-transformed model)

**Figure 3:**
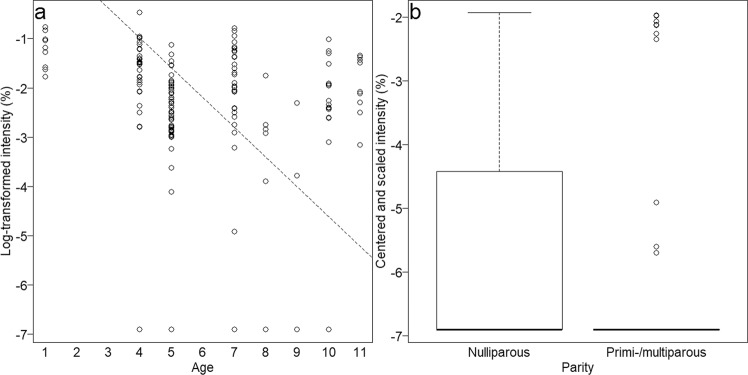
Influence of age (**a**: 2,5-dimethyl-pyrazine – same as for normalised model) and parity (**b**: methenamine) on log-transformed intensities of the most affected substances. Boxplot shows medians as well as first and third quartiles and the dashed line in scatterplot shows random slope estimates derived from a model with the fixed effects centred to a mean of zero.

The log-transformed model revealed substances which were affected by age and parity. Accordingly, their intensities changed relative to the rest of the chemical profile and thus, need to be interpreted by a receiver in the context of the whole profile. The substance which showed the strongest change with age was identified as 2,5-dimethyl-pyrazine (see Fig. 3a), which also had the largest age-dependent change in the normalised model. Together with 2,5-dimethyl-pyrazine, five age-dependent substances decreased and three substances increased with age (see Table 3). Similar to the normalised model, most of the affected substances for parity (see Table 3) were higher in nulliparous females, including methenamine (see Fig. 3b) with the strongest intensity change. Only two substances were higher in primi-/multiparous females.

## Discussion

Our study combined behavioural bioassays with chemical analyses in common marmosets to investigate female fertility cues. We confirmed and expanded existing behavioural evidence that male common marmosets can use odours to identify the peri-ovulatory cycle state of females. Furthermore, we narrowed the gap between evidence from behavioural observations and the underlying chemical profiles by showing that the chemical composition of body scents varies with female fertility of common marmosets. As predicted, cycle states, age and parity had independent influences on the composition of chemical profiles.

Complementing the chemical focus of the study, the bioassay results confirmed and expanded on behavioural bioassays from other studies on marmosets (see refs^23,30^). Smith and Abbott previously showed that male marmosets discriminate between scents from females in the peri-ovulatory and the luteal phase^23^. We also found that seven of eight male marmosets sniffed and marked longer at swabs from the peri-ovulatory than from the luteal phase. Additionally, we showed that those seven males also distinguished between the peri-ovulatory and the follicular phase. Hence, males seem to be able to discriminate females during their peri-ovulatory phase from females during their non-ovulatory phase by their scents. A longer investigation of one scent sample compared to another sample could indicate that this one is preferred over the other, less familiar, of lower intensity or harder to decipher^53^. However, in the present context we consider it likely that the peri-ovulatory phase evoked greater interest compared to the follicular and the luteal phases in order to detect the receptive female.

While this pattern was clear in all but one of the males we found only weak statistical support for this ability to discriminate due to one male investigating both non-peri-ovulatory samples longer than the respective peri-ovulatory samples. This male distinguished between a blank sample and a female sample in the initial test, suggesting that his responses were not due to an inability to smell. There were also no obvious differences in the attributes of this male compared to the other males. The presented female scents evoked no unusual responses in any of the other males, nor did the donors of preferred scents differ systematically in age, rank or familiarity from the donors of non-preferred scents or other females in this study. Furthermore, the male’s cage mate was not related to the donor females and it was not in the peri-ovulatory phase during the two discrimination tasks.

Our chemical analysis revealed that cycle states were associated with the similarity of entire chemical profiles and, in addition, affected the chemical composition in the normalised model, but not in the log-transformed model. Differences in the chemical composition could reflect a change of cycle-dependent substances over the menstrual cycle, which needs to be tracked over time and considered independently from the relation to the rest of the chemical profile (normalised model). Alternatively, they could reflect a relative change of cycle-dependent substances within a chemical profile specific for a cycle state (log-transformed model). The latter would allow males to identify the menstrual cycle state of a female by detecting a fertility-specific chemical composition of scents without tracking the females’ scents over the course of her menstrual cycle, as it was required in the bioassay tests. Under natural conditions, this recognition of a fertility-specific chemical composition would be used when spotting a scent mark left in the environment from an unfamiliar female. Indeed, the bioassay results of this and previous studies^23^^,^^30^ suggest that males can derive information about cycle states in this manner, which is also in line with the fertility-associated similarity of entire chemical profiles. As such, we also would have expected a change of substance intensities with the log-transformed model, which focused the analysis on changes in substance intensities relative to the rest of the chemical profile at a single point in time. This discrepancy between behavioral and chemical results could possibly be due to the more sensitive odor perception of animals, which, in contrast to our analytical and statistical tools, is specifically tuned to detect relevant cues. In this case, an expansion of the study with a higher number of sampled females could facilitate pinpointing potential subtle changes in olfactory cues amongst the multitude of substances that make up odor profiles. Nonetheless, we detected changes of substance intensities in the normalised model, which can be interpreted as differences between different time points of the menstrual cycle rather than differences in relation to the rest of the chemical profile at a specific point in time (as in the log-transformed model). Such differences would require males to keep track of a female’s scents over her menstrual cycle, i.e. during daily contact with the breeding female. Such temporal changes could initially be used, for example, by unexperienced males to learn the fertility-specific chemical patterns. This aspect was not addressed in the current or previous bioassays and constitutes a valuable addition to future bioassay studies.

For all substances that were considerably affected by cycle states in the normalised model (i.e. absolute slope estimate larger than two standard deviations above average slope estimate) the absolute difference between the peri-ovulatory and the luteal phase was larger than between the follicular and the peri-ovulatory phase. The highest substance intensities were noted not only for the peri-ovulatory but also for the luteal phase, as we predicted. Assuming that the chemical difference is driven by a decomposition of cycle-related hormones, it is reasonable that the main cycle-dependent effect is seen within or after the peri-ovulatory phase, because these phases coincide with the degradation of ovulation-specific hormones. Such cues would allow marmoset males to restrict their mating efforts to the peri-ovulatory phase and not beyond that period. Indeed, mating increases within the period of likely conception in wild common marmosets^28^ and we, as well as a previous study, showed that captive male marmosets are able to detect the olfactory difference between the peri-ovulatory and the luteal phase^23^.

The comparison of substance intensity across cycle states (suggested by the normalised model) revealed substances from various chemical classes (aromatic hydrocarbons, an alcohol, an ester and a heterocyclic compound). While no cycle-dependent substances have been identified in marmosets up to now, studies in other mammals reported cycle-dependent carboxylic acids (*Macaca mulatta*^54^), alcohols and alkanes (*Antelope cervicapra L*.^7^) as well as aromatic hydrocarbons (*Equus caballus L*.^55^) based on urine or genital secretions. Our marmoset samples are likely to contain substances originating from genital secretions, but also from so called ‘specialised’ apocrine and sebaceous glands as well as ‘non-specialised’ skin glands, urine and faeces which are also present in the genital area^23^. Subsequently, the exact origin and metabolic pathway of those substances should be examined to understand the underlying mechanisms and to clarify whether any of them evolved to signal the fertility of females.

Age and parity are often confounded with each other as older females most likely gave birth to offspring more often than younger females. However, we were able to examine both factors separately given our study design and found independent influences on the chemical profiles. Age relates to a certain physical condition^56^ and endocrine functions^57^ and thus, should have an effect on chemical profiles. Indeed, we found age to be associated with the overall similarity of chemical profiles as well as the chemical composition in both, the normalised and log-transformed model. Thus, age-dependent substances seem to vary both relative to the whole profile and across time.

A previous study on great apes^33^ suggested more substances to decrease than to increase with increasing age, leading to the expectation that age-dependent substances in the present study would also decrease over time. Indeed, most age-dependent substances of both models were decreasing (substance classes: pyrazine, alcohol, aldehyde, ester, steroid, ketone, carboxylic acid and aromatic hydrocarbon), but also few were increasing (substance classes: aromatic hydrocarbon, ester and aldehyde) with increasing age. This is in accordance with other studies which also describe decreases as well as increases of substances with age belonging to various substance classes (aldehydes, alcohols, ketones, alkanes, amides, aromatic hydrocarbons, carboxylic acids, steroids and heterocyclic compounds, ester) when examining chemical profiles of urine, body odour or faeces (*Mus spec*.^58^, humans^59^, *Ceratotherium simum*^60^, *Macaca mulatta*^61^). In addition, various bioassay studies confirmed that age differences in chemical profiles of different species (e.g. humans^38^) are detectable by human raters. In common marmosets, age seems to negatively affect the reproductive output of females^62^, (whereas no effect was found in ref.^63^). Thus, an olfactory cue of age could enable males to choose younger (and more fertile) females as indicated by a study on crab eating macaques (*Macaca fascicularis*^64^), for example, when a new breeding female needs to be selected.

Parity, on the other hand, measured as nulliparous vs. primi-/multiparous in this study, seems to represent maternal experience^56^, with nulliparous females facing a high risk of losing their first litter even in social breeders (*Callithrix jacchus*^63^). Thus, males could prefer a more experienced female as breeding partner to enhance their own reproductive success, particularly if they can perceive such information via olfactory cues. In mandrills (*Mandrillus sphinx*), males mate-guard parous females more often than nulliparous females probably to choose the more experienced female^65^. However, there have been no studies examining the influence of parity on chemical profiles by comparing nulliparous and primi-/multiparous females until now. Our study revealed an effect of parity on the chemical composition in both the normalised and the log-transformed model, but no association with the overall similarity of chemical profiles. For both models, most of the affected substances had higher intensities in nulliparous than in primi-/multiparous females (classified as ester, aromatic hydrocarbon, alcohol, ketone, heterocyclic compound, steroid, pyrazine and aldehyde). Only two substances were more abundant in primi-/multiparous females (classified as ester and aromatic hydrocarbon) following our prediction of changes in line with ‘mammary chemosignals’^34^.

Although we intended to find endogenous olfactory cues of fertility, some of the parity-related substances seem to be of non-mammalian origin (e.g. diisobutyl phthalic acid ester, methenamine). Substances of non-mammalian origin found in chemical profiles could include degradation products of bio-accumulated contaminants. A study in humans^66^ reported an off-load of contaminants from mothers to their first offspring, accumulated from polluted environment and food. An excretion of environmental chemicals within breast milk during lactation was shown for humans^67^ and even phthalates, as we found here, were detected in breast milk^68^. Thus, the decrease of some of the substances could be caused by elimination of accumulated contaminants during lactation. For the remaining endogenous substances, future studies should examine which physical or hormonal changes induce parity-related changes in the chemical profile.

We confirmed and expanded on previous behavioural studies demonstrating that common marmoset males are able to detect fertile states of females by olfaction. Moreover, we narrowed the gap of whether these olfactory cues are locatable in the chemical profiles. We showed that fertility differences exist in the chemical profile of female marmosets based on several characteristics. An overall chemical profile of an individual consists of a few hundred substances which encode a wealth of information on either stable or transient traits, such as individual identity, sex, age, rank and species identity (reviewed in ref.^17^). Our results show that fertility is encoded in chemical profiles. Identifying substances accounting for changes in chemical profiles provides the basis for comparative studies across non-human primates to unravel the importance of chemical fertility cues during primate evolution. Follow-up studies should subsequently confirm the identification of the substances identified in this study with chemical standards, investigate if males respond to these specific substances (i.e. bioassays with mixtures of chemical standards reconstructing fertility cues), discover the metabolic origin of these substances and assess whether they evolved to advertise the fertile state of females.

## Supplementary information

Dataset 1

## Data Availability

The datasets generated and analysed during the current study are available on Zenodo: http://doi.org/10.5281/zenodo.1341204. Self-written R scripts are available from the authors upon request.
